# *Borrelia crocidurae* Infection in Acutely Febrile Patients, Senegal

**DOI:** 10.3201/eid2008.130550

**Published:** 2014-08

**Authors:** Oleg Mediannikov, Cristina Socolovschi, Hubert Bassene, Georges Diatta, Pavel Ratmanov, Florence Fenollar, Cheikh Sokhna, Didier Raoult

**Affiliations:** Aix-Marseille Université, Marseille, France, and Dakar, Senegal (O. Mediannikov, C. Socolovschi, H. Bassene, G. Diatta, P. Ratmanov, F. Fenollar, C. Sokhna, D. Raoult);; Far Eastern State Medical University, Khabarovsk, Russia (P. Ratmanov)

**Keywords:** Borrelia, crocidurae, relapsing fever, Senegal, fever, bacteria

## Abstract

As malaria cases in Africa decline, other causes of acute febrile illness are being explored. To determine incidence of *Borrelia crocidurae* infection during June 2010–October 2011, we collected 1,566 blood specimens from febrile patients in Senegal. Incidence was high (7.3%). New treatment strategies, possibly doxycycline, might be indicated for febrile patients**.**

As malaria cases decline, the need for programs to study the roles of other causes of febrile syndromes in Africa increases (*1*). Other causes of fever in outpatients include typhoid and paratyphoid fevers; pneumococcal bacteremia; and a spectrum of viral infections, including influenza, yellow fever, dengue, chikungunya, and Rift Valley fever (*2*). In preliminary studies, we detected the DNA of *Borrelia crocidurae, Rickettsia* spp., *Tropheryma whipplei*, and *Coxiella burnetii* in environmental samples and blood specimens from febrile patients in Senegal (*3*–*6*).

In western Africa, tick-borne relapsing fever (TBRF) is caused by *B. crocidurae*; this acute febrile illness produces multiple recurrences of nonspecific signs and symptoms, including fever, headache, myalgia, and arthralgia. After decades of neglect, TBRF has again been detected (*7*) and is thought to be one of the major causes of fever in Africa. The reported incidence rate for TBRF in western Africa is high, reaching 25 cases per 100 person-years or accounting for 13% of febrile illnesses treated at rural dispensaries (*4*,*8*). This incidence rate is even higher than that for TBRF caused by *B. duttonii* in eastern Africa (*9*). A study from Tanzania found *B. duttonii* DNA in 3.9% of blood samples (*10*).

In 2008, we began to create a network of rural dispensaries from which to recruit patients (Table; Figure 1). The 5 study sites covered several ecosystems, ranging from dry Sahelian in northern Senegal (Keur Momar Sarr, Niakhar, and Sine-Saloum) to humid sub-Guinean in southern Senegal (Casamance and Kedougou). Two seasons are typical: dry (November–May) and rainy (June–October). The National Ethics Committee of Senegal approved the study (*11*,*12*). Since 2010, the populations of both villages in Sine-Saloum (Dielmo and Ndiop) have benefited from routine rapid point-of-care laboratory diagnostics (*12*). 

**Table:** Rural health centers and laboratories participating in study of *Borrelia crocidurae* infection in acutely febrile patients, Senegal, June 2010–October 2011*

Study site	District/ dept/ region	District pop	Climate/ vegetation	Precip, mm/y	Health center	Coord	Other	IR
Sine-Saloum	Toubacouta/ Foundiougne/ Fatick	120,554	Sudanian/wooded savannah	939	Dielmo	13°43′N, 16°24′W	POC	10.5 (70/669)
					Ndiop	13°41′N, 16°23′W		
Niakhar	Niakhar/ Fatick and Niakhar/ Fatick	69,446	Sahelo-Sudanian/wooded steppe	757	Toucar	14°32′N, 16°28′W		19.1 (33/173)
					Diohine	14°30′N, 16°30′W		
					Ngayokheme	14°32′N, 16°26′W		
					Niakhar†	14°28′N, 16°23′W	DNA extraction	
Casamance	Loudia-Oulof/ Oussouye/ Ziguinchor	57,505	Sub-Guinean/primary and secondary gallery forests	1,432	Mlomp	12°33′N, 16°34′W	DNA extraction	0.6 (2/315)
					Kagnout	12°33′N, 16°37′W		
					Elinkine	12°30′N, 16°39′W		
Kedougou	Bandafassi/ Kedougou/ Kedougou	20,021	Sudano-Guinean/woodland, wooded savannah	1,189	Bandafassi	12°32′N, 12°18′W	DNA extraction	0.4 (1/246)
					Ibel	12°30′N, 12°22′W		
					Tiabeji	12°38′N, 12°25′W		
Keur Momar Sarr	Keur Momar Sarr/Louga/ Louga	70,743	Sahelian/steppe type	400	Keur Momar Sarr	15°55′N, 15°58′W	DNA extraction	5.5 (9/163)
					Loboudou	15°57′N, 15°55′W		
					Ganket Balla	15°58′N, 15°55′W		

*Climate information obtained from http://www.au-senegal.com/IMG/png/climat.png; demographic data obtained from the National Agency of Statistics and Demography of Senegal (http://www.ansd.sn). dept, department; pop, population; precip, precipitation; coord, coordinates; IR, *B. crocidurae* incidence rate, % (no. tested/no. positive); POC, point-of-care laboratory. †No patient recruitment at this site.

**Figure 1:**
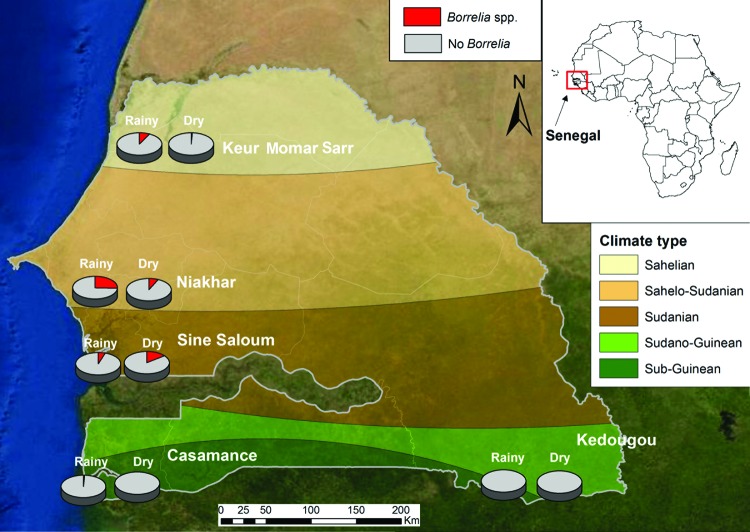
Incidence of tick-borne relapsing fever at study sites, according to season, Senegal, June 2010–October 2011. Map reproduced from Bing Maps (http://www.bing.com/maps/).

## The Study

From June 2010 through October 2011, fingerstick blood samples (200 μL) were collected from 1,549 febrile patients (axillary temperature >37.5°C) at 14 dispensaries and 91 randomly selected healthy villagers in Senegal. Samples were subjected to DNA extraction. Digesting, binding, and washing were performed directly in the village dispensaries by use of the QIAamp kit (QIAGEN, Hilden, Germany) as previously reported (*6*). DNA elution was performed at Aix-Marseille Université in Marseille, France. Quantitative PCR (qPCR) was performed by using primers and probes specific for the genus *Borrelia*. All samples positive for *Borrelia* spp. were subjected to standard PCR (*flaB* gene) (*4*). To determine the quality of the extracted DNA, we also measured the human actin gene (*5*). The samples were considered positive only if qPCR and *flaB*-based PCR results were positive; the sequencing of all *flaB* amplicons demonstrated that they belonged to *B. crocidurae*. 

Isolation of borreliae involved intraperitoneal inoculation of laboratory BALB/c mice with 100 μL of patient capillary blood. Borreliae in mice were detected by microscopic examination of Giemsa-stained peripheral blood smears followed by qPCR of blood samples. 

The sex ratio for the 1,549 patients did not differ significantly among sites (772 male and 777 female patients). An analysis of 6 age groups (<12 months, 1–3 years, 4–6 years, 7–15 years, 16–29 years, and >30 years) showed no differences among sites. All tested samples from clinically healthy persons had negative qPCR results for borreliae.

The incidence rate was calculated as the number of febrile episodes divided by the person-time multiplied by 1,000 (data available only for the Sine-Saloum site). The incidence rate of febrile episodes was 0.80 in Dielmo and 0.36 in Ndiop (p<0.05). Among the 1,566 samples tested, 115 (7.3%) were positive for *B. crocidurae.* The incidence rate for TBRF was 9.7 cases/100 persons in Dielmo and 2.4 cases/100 persons in Ndiop. The first autochthonous cases in Ndiop, which was previously considered borreliosis free, were observed in October 2010; incidence was significantly lower in Ndiop than in Dielmo (p<0.05). All cases registered in Ndiop before October 2010 were included in the epidemiologic investigation and considered to be imported. The proportion of the *Borrelia*-positive samples was significantly higher for northern sites with a drier Sudanian climate; positivity reached 19.1% (33/173) in Niakhar (Table). By analyzing the epidemiologic questionnaires completed by families of ill persons, we determined that the 2 TBRF cases in Casamance were imported from the northern regions of Senegal by seasonal workers.

Patients most frequently infected were 7–15 years of age (13.5%, 43/318), unlike in eastern Africa where younger persons are more frequently infected (*9*). No positive results were found among the 155 children <12 months of age, but positive results were found for 16 (4.8%) of the 352 children 1–3 years of age (p<0.05). Unlike in other northern regions, in Sine-Saloum, the proportion of *Borrelia*-positive samples was significantly higher for samples collected during the dry (16.9%, 40/237) than the rainy season (6.9%, 30/432); p<0.0001 (Figure 1).

**Figure 2:**
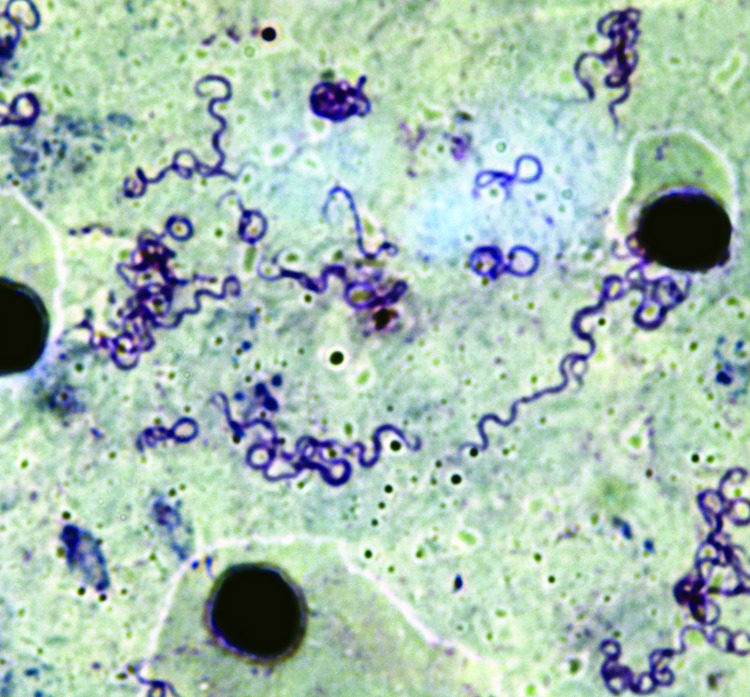
Thick smear of mouse blood showing *Borrelia crocidurae* isolate 02–03. Giemsa stain; original magnification x900.

We identified 20 patients (49 samples) for whom 2–4 samples were positive for *B. crocidurae*. The interval between the sample collections was short (5–30 days) for 13 persons, average (30–66 days) for 4 persons, and long (102–381 days) for 3 persons. Two *Borrelia* isolates (no. 03–02 from Ndiop and no. 19/31 from Dielmo) were recovered from the peripheral blood of 2 febrile patients. The bacteria had a morphologic appearance that was typical for borreliae (Figure 2). A BLAST (http://blast.ncbi.nlm.nih.gov/Blast.cgi) search for the sequenced *flaB* gene (JX119098) demonstrated that the isolates were nearly identical with the type Achema strain of *B. crocidurae* (CP003426).

## Conclusions

We detected an alarmingly high proportion of *Borrelia* DNA in the blood of febrile patients in Senegal. The presence of this DNA is strongly and specifically linked to the fever because no *Borrelia* DNA was identified among the 90 control participants. In Tanzania, however, borreliae have been identified in up to 33% of blood samples obtained from asymptomatic blood donors who lived in similar conditions as ill persons (*13*).

The geographic repartition of TBRF is linked to drier climates (*9*). We observed autochthonous cases only in northern Senegal, roughly north of the 13°30′ parallel. We noted the recent extension of *B. crocidurae* into the village of Ndiop, which had been free of *B. crocidurae*. This extension might be linked to recent climate changes (*14*). The person-year incidence of borreliosis in our study (6.1 cases/100 population) is similar to that reported by Vial et al. (4 cases/100 population) for the interepidemic period (*8*).

We report a unique series of cases in which *Borrelia* DNA was identified several times consecutively in the blood of the same patient. For 17 patients for whom the time between positive samples was short or average (up to 66 days), repeated detection of *Borrelia* DNA during repeated episodes of fever could be explained by relapses. However, reinfection is strongly suspected in 3 patients because the interval between 2 positive samples was >100 days. To the best of our knowledge, reinfection with relapsing fever borreliae has not been previously reported in Africa. The phenomenon of easy reinfection after treatment with tetracycline has been reported for the relapsing-fever group *B. hermsii* in vervet monkeys, which could be reinfected 12–36 weeks after primary infection (*15*).

In conclusion, the incidence of TBRF and the proportion of borreliosis cases among febrile patients in Senegal is very high and, in at least 1 region (Niakhar), exceeds that of malaria. This considerably high incidence rate should lead to the development of new therapeutic strategies that could be based on treating febrile patients in Senegal with doxycycline.
